# A Simplified and Efficient Process for Insulin Production in *Pichia pastoris*

**DOI:** 10.1371/journal.pone.0167207

**Published:** 2016-12-01

**Authors:** Sulena Polez, Domenico Origi, Sotir Zahariev, Corrado Guarnaccia, Sergio G. Tisminetzky, Nataša Skoko, Marco Baralle

**Affiliations:** 1 ICGEB, Trieste, Italy; 2 Biomanufacturing Sciences Network, Process Solutions, Merck SpA, Vimodrone (Milan), Italy; Weizmann Institute of Science, ISRAEL

## Abstract

A significant barrier to insulin is affordability. In this manuscript we describe improvements to key steps in the insulin production process in *Pichia pastoris* that reduce cost and time. The strategy for recovery and processing of human insulin precursor has been streamlined to two steps from bioreactor to the transpeptidation reaction. In the first step the insulin precursor secreted during the methanol induction phase is recovered directly from the culture broth using Tangential Flow Filtration with a Prostak^™^ module eliminating the laborious and time-consuming multi-step clarification, including centrifugation. In the second step the protein is applied at very high loadings on a cation exchange resin and eluted in a mixture of water and ethanol to obtain a concentrated insulin precursor, suitable for use directly in the transpeptidation reaction. Overall the yield from insulin precursor to human insulin was 51% and consisted of three purification chromatography steps. In addition we describe a method for recovery of the excess of H-Thr(tBu)-OtBu from the transpeptidation reaction mixture, one of the more costly reagents in the process, along with its successful reuse.

## Introduction

Insulin is a relatively low-priced drug however, the chronic nature of Diabetes means the cost for insulin treatment is high, and together with an increasing number of patients, this financial burden challenges healthcare systems worldwide. Its price reduction is needed in order to improve its availability especially in lower to lower-middle income countries [1].

Two major pathways for large-scale production of recombinant human insulin are currently used [2]. One route uses *E*. *coli* as an expression host, where the overexpressed insulin precursor (IP) forms inclusion bodies requiring solubilisation and oxidative refolding. The second route uses yeast-based expression systems (mainly *Saccharomyces cerevisiae*) where the IP is directly secreted in the culture supernatant in its correctly folded conformation. Half of the world insulin supply derives from *Saccharomyces cerevisiae* [3]. However, the standard recovery and purification process of human insulin produced in *Saccharomyces cerevisiae* can include up to fifteen steps [4].

The methylotrophic yeast *Pichia pastoris* has shown a number of attractive characteristics for heterologous protein production in virtue of its capacity of reaching high cell densities and expressing high amounts of recombinant proteins under control of strong and tightly regulated promoters [5]. Using this system we have previously described a simple two-phase cultivation process composed of a glycerol batch and a constant methanol fed-batch phase for secretory IP production resulting in a ~2 fold enhancement of IP production compared to the highest values reported, significantly increasing the efficiency of insulin manufacture [6].

In this manuscript whilst a simplified fed-batch fermentation protocol for IP production, we developed an optimized and scalable strategy employing a small scale Prostak^™^ prototype Tangential Flow Filtration (TFF) system to recover IP from culture broth eliminating the laborious steps of broth centrifugation and secondary clarification of the supernatant. We subsequently developed a novel and efficient chromatographic procedure to purify the IP using a cation exchange resin at high loading capacity and performing IP elution in a water/ethanol mixture that is then readily applicable in the transpeptidation reaction without intermediate steps. These modifications reduced the entire insulin manufacturing process, from culture supernatant to the final human insulin, to seven steps. The solutions presented here reduce the process time and have significant repercussions on financial and operational plans in scale-up procedure, bypassing multiple freeze-drying steps within the process.

## Materials and Methods

### Strains and vectors

The IP pPIC9K expression vector construction was performed as previously described [6]. The expression plasmid pPIC9K-IP was linearized with restriction enzyme *BglII* and electroporated into *P*. *pastoris* strain GS115his^-^ (Invitrogen). Transformants were selected on plates with increasing concentration of antibiotic G418 in order to select multicopy clones (Pichia protocols, Invitrogen). Selected clones were grown in liquid culture and the culture supernatant was tested for IP production by SDS-PAGE and RP-HPLC. The best producing clone was grew on 2 g L^-1^ of G418. Methanol utilization phenotype determination was performed by standard protocol plating the colonies on Minimal Dextrose (MD) plates and Minimal Methanol (MM) plates. The selected clone grew normally on MD plate but show little growth on the MM plate indicating its Mut^s^ (methanol utilization slow) phenotype, where the AOX1 gene is knocked out.

### Culture growth conditions and IP production

A pre-culture was made by inoculating 100 ml YPG with a single colony from the yeast extract peptone dextrose plate with 2 g L^-1^ G418 and incubating for 24 hours at 30°C. This was then diluted to 1.2 L and incubated as above. The pre-culture seed was then transferred to the 30 L Sartorius BIOSTAT Cplus-C30 bioreactor (Sartorius) containing 15 L of autoclaved growth media, adjusted to pH 5.0 with 25% NH_4_OH. The growth media composition was: glycerol 62.5 g L^-1^, H_3_PO_4_ 26.7 ml L^-1^, CaSO_4_x 2H_2_O 0.93 g L^-1^, K_2_SO_4_ 18.2 g L^-1^, MgSO_4_ x 7H_2_O 14.9 g L^-1^, KOH 4.13 g L^-1^. Prior to inoculation, 4 ml of Biotin 0.2 g L^-1^ and 4 ml of PMT1 were added to the growth media. PTM1 composition: CuSO_4_ x 5H_2_O 6 g L^-1^, KI 0.08 g L^-1^, MnSO_4_ x H_2_O 3 g L^-1^, H_3_BO_3_ 0.02 g L^-1^, CaSO_4_ x 2H_2_O 0.5 g L^-1^, ZnSO_4_ x 7H_2_O 35.59 g L^-1^, H_2_SO_4_ 5 ml L^-1^ FeSO_4_ x 7H_2_O 65 g L^-1^, CoCl x 6H_2_O 0.5 g L^-1^, (NH_4_)_6_Mo_7_O_24_ x 4H_2_O 0.2 g L^-1^ [7]. Aeration rate was maintained at 1.5 vvm with inlet gases (air and oxygen) and stirrer speed was controlled between 200 to 800 rpm in order to maintain a dissolved oxygen (DO) concentration of 35% constant throughout the process. Temperature was maintained at 30°C and pH at pH 5.0 with 25% NH_4_OH or H_3_PO_4_. Antifoam agent utilised was Schill and Schelinger's Struktol J673A [8].

After 23 hours, when all glycerol was depleted, as indicated by an increase of the DO concentration, glycerol-feeding phase with glycerol solution (50% glycerol (w/w), 4 ml L^-1^ biotin solution 0.2 g L^-1^ and 4 ml L^-1^ PTM1) at speed 15 ml L^-1^ h^-1^was initiated. After 6 hours, production of IP was initiated by step-wise addition of a methanol solution (99.2% (w/w) methanol and 4 ml L^-1^ Biotin 0.2 g L^-1^ and 4 ml L^-1^ PTM1). In the first 6 hours methanol addition speed was 1.1 ml L^-1^ h^-1^. Subsequently flow rate was increased to 6.6 ml L^-1^ h^-1^ and maintained constant for the next 6 days. Insulin precursor (IP) quantification was performed via analytical RP-HPLC employing a column Vydac 214 MS C4 (4.6x250mm, 5μm). Mobile phase A was 0.1% (v/v) TFA in water and elution was done at 25°C with a gradient of mobile phase B (0.1% TFA (v/v) in acetonitrile) as follows: 0–20%B (0–6 min), 20–46%B (6–32 min). The flow was 1 ml/min and the column effluent was monitored at 280 nm. A calibration curve with the human insulin CRS from European Pharmacopoeia (EP) was made in order to extrapolate the amount of insulin precursor and other insulin species.

### Recovery of ip from culture broth with tangential flow filtration

Culture broth pH was adjusted to pH 3.0 with 85% phosphoric acid. The culture broth (4 L) was clarified in a small scale Prostak^™^ prototype Tangential Flow Filtration system (Merck Millipore). The prototype module incorporates the Durapore 0.22 μm membrane (PVDF) and has a surface area 0.063 m^2^. A secondary pump placed on the device’s permeate port, in order to control the permeate extraction rate and to avoid possible membrane’s premature plugging due to the high intrinsic permeability typically exhibited by microfiltration membranes at filtration start.

Concentration of the sample was then run at a permeate extraction rate of 27.5 LMH, 74% of the Critical Permeate Flux (CPF). The concentration was stopped at the volume concentration factor (VCF) of 2.7X. Diafiltration was performed, with Milli-Q grade water, in a cyclic manner by adding a volume corresponding to the concentrated harvest, thus doubling the initial feed volume, and subsequently reducing the feed volume to the original level (discontinuous mode). This was performed in order to avoid high pressure (inlet pressure and TMP as high as, respectively, 1.5 bar and 0.8–1 bar), and thus to cyclically relax the polarization layer due to the high solids content reached following concentration. Diafiltration volume was 6.5 L, therefore total extracted volume (permeate and diafiltrate) containing IP molecule was 9L. For turbidity measurements HACH portable turbidimeter (0 ¨C 1000 NTU) was used.

### IP purification and concentration

Purification of IP was performed by cation exchange chromatography using Toyopearl GigaCap S-650 M resin (Tosoh Bioscience) or Sepharose SP Fast Flow resin (GE Healthcare). 110 mg of IP was loaded for each ml of Toyopearl GigaCap S-650 M resin (column volume 105 ml) directly from the TFF system. Conductivity of the sample was 15 mS/cm. Mobile phase A: 20 mmol L^-1^ NaOAc, pH 4.0, mobile phase B was: 20 mmol L^-1^ NaOAc, pH 4.0, EtOH 50%, and mobile phase C: 20 mmol L^-1^ NaOAc, pH 4.0, EtOH 50%, NaCl 0.5 mol L^-1^. Sample was loaded on the column pre-equilibrated in mobile phase A with flow rate of 137.6 cm h^-1^. After sample loading, the column was washed with 3 column volumes of mobile phase A. Flow rate was decreased to 45.9 cm h^-1^and elution was initiated with mobile phase B for 3.5 column volumes. The bound IP was eluted from the column with the mobile phase C with the flow rate of 45.9 cm h^-1^. The majority of IP was eluted in the first column volume and the concentration obtained was 80 mg ml^-1^.

### Transpeptidation reaction, deprotection and purification of human insulin

IP eluted in 20 mmol L^-1^ NaOAc, pH 4.0, EtOH 50%, NaCl 0.5 mol L^-1^ was directly subjected to transpeptidation. The transpeptidation reaction was performed in a mixture containing 2.5 g of IP (8.27 mmol L^-1^), 0.1 g porcine trypsin (1/25, w/w in respect to IP, 0.08 mmol L^-1^), 7 μmol L^-1^ CaCl_2_, 0.4 mol L^-1^ H-Thr(tBu)-OtBu.AcOH, 37.5% dimethylformamide, with the pH adjusted to 7.0 with Tris base. The reaction was performed at 20°C for 24 hours.

The reaction was stopped by diluting the mixture with 100 mmol L^-1^ NaOAc buffer, pH 4.0 to the final IP concentration of 2 g L^-1^ and loaded onto PLRP-S 300Å 8 μm resin (Agilent Technologies). The bound insulin ester was eluted with a flow rate of 62.4 cm h^-1^ by a gradient formed by mixing solutions A (0.2 mol L^-1^ ammonium sulphate buffer, pH 2.0) and B (0.1 mol L^-1^ ammonium sulphate buffer with 40% (v/v) 2-propanol) as follows: 0–60% B, 60% B (2.3 column volumes, CV), 60–75% B (4 CV), 75% B (0.5 CV), 75–81% B (4.7 CV). Insulin ester elutes from the column during the last gradient step.

The purified insulin ester was crystallized by addition of 2 mg of ZnCl_2_ for each mg of pre-insulin, adjusting to pH 6 and incubation at +4°C for 16 hours. Insulin ester crystals were recovered by centrifugation at 5000 g for 20 min at +4°C (Sorvall RC 5B plus centrifuge, SLA3000 rotor) and drying at room temperature. Insulin ester was converted into human insulin by removing the tertiary-butyl groups of threonine in position B30 by acidolysis with trifluoroacetic acid (deprotection reaction) as described before [6]. After 60 minutes of deprotection, the reaction mixture was diluted ro 1mg/ml in 50 mmol L^-1^ NaOAC and pH adjusted to 2.8 for loading on PLRP-S 300A 8 μm resin. The elution used 50 mmol L^-1^ Na_2_SO_4_ + 10% 2-propanol + 2% acetic acid as mobile phase A and 50 mmol L^-1^ Na_2_SO_4_ + 50% 2-propanol + 2% acetic acid as mobile phase B in a gradient mode: 0–18% B (0.1 CV), 18% B (1 CV), 18–38% B (7.8 CV), 38–100% B (0.5 CV). Human insulin elutes from the column in the 18–38% B gradient step.

All reagents were purchased from Sigma, except: H-Thr(tBu)-OtBu.AcOH (Unibiochem), Tris (Invitrogen), dimethylformamide (Romil), CaCl_2_ (Merck). Porcine trypsin was purchased from Sigma (13000–20000 BAEE U/mg, T0303).

### Extraction of the excess of H-Thr(tBu)-OtBu.AcOH

The flow-through volume up to the first protein peak from the preparative reversed-phase chromatography (RP-HPLC, post-transpeptidation) was collected and confirmed, by analytical RP-HPLC and Evaporative Light Scattering Detector (Alltech 3300 ELSD by Grace, Columbia, Maryland, USA), to contain H-Thr(tBu)-OtBu. From this fraction we recovered H-Thr(tBu)-OtBu by water organic solvent extraction [9]. 1.8 L of “flow-through to first peak” fraction was mixed with 150 g Na_2_CO_3_ and 50 g NaCl. H-Thr(tBu)-OtBu was extracted with 200 ml and then three times with 100 ml of EtOAc/Et2O (1/1, v/v). Upper layers containing H-Thr(tBu)-OtBu were collected and back extracted with 100 ml of saturated solution of NaCl. Furthermore, collected organic layer containing H-Thr(tBu)-OtBu was dried with 22 g of Na_2_SO_4_, filtered and insoluble Na_2_SO_4_ on the filter was washed twice with 30 ml EtOAc. Solvent was removed under reduced pressure. The rest (clear oil) was dried at room temperature in vacuum at 0.5 mbar residual pressure to constant weight (around 24 hours). The purity of extracted H-Thr(tBu)-OtBu was confirmed by analytical RP-HPLC analysis on a column Gemini 5RP18 4.6x150 mm (Phenomenex). The injected sample was around 0.06 mg. Mobile phase A was 0.1% (v/v) TFA in water and elution was performed at 1ml/min with a gradient to mobile phase B (0.1% TFA (v/v) in acetonitrile) as follows: 5% (0–1 min), 5–100% B (1–31 min). Retention time of H-Thr(tBu)-OtBu was 12.36 min. and the column effluent was monitored by UV at 214 nm and by ELSD detector.

### Mass spectrometry

Electrospray mass spectrometry (ESI-MS) analysis was performed with an ion trap mass spectrometer (amaZonSL, Bruker Daltonics). Samples were either reconstituted in 0.1% formic acid in 50% acetonitrile and directly infused or analyzed by LC-MS using 0.1% formic acid/water and acetonitrile mobile phases. Spectra were acquired in positive ion mode using Compass Trap Control software and deconvoluted, to obtain monoisotopic or average masses, using Compass Data Analysis software (Bruker Daltonics).

## Results

### Production of Insulin precursor in fed-batch fermentation

Human IP nucleotide sequence was codon-optimized for expression in *Pichia pastoris* and chemically synthesized and constructed as previously described [6]. N-terminal extension of the insulin precursor (EEAEAEAEPK) was employed as this has been shown to increase the Kex2 endoprotease efficiency and the fermentation yield of IP [10]. The IP gene was fused in frame with the *S*. *cerevisiae* α-factor signal sequence in the integrative vector pPIC9K. The best IP producing clone of transformed *P*. *pastoris* GS115 strain was selected on G418 containing plate (2 g L^-1^) and was found to be Mut^s^. High-density fermentation was performed in 30 L bioreactor using 15 L of culture media as described in material and methods. This simplified fermentation method with no need of a methanol detector and constant in-process methanol concentration determination resulted in average of 308 g L^-^ (calculated from 3 fermentations) of wet cell weight and an expression level of IP in the culture supernatant of 2.26 g L^-1^. The culture supernatant from bioreactor during 6 days of methanol induction was subjected to mass spectrometry analysis and insulin precursor species with different length of N-terminal extension EEAEAEAEPK were detected (S1 Fig). This was relatively unexpected as the use of N-terminal extension of the insulin precursor (EEAEAEAEPK) has been shown to increase the Kex2 endoprotease efficiency and the insertion of Glu before the N-terminal Glu to inhibit cleavage by Dipeptidyl aminopeptidase A (DPAPA) [10]. Moreover, in this study N-terminal extension was observed to be protective against the action of the Yap3p by insertion of a Pro before Lys, without affecting the efficiency of the Kex2 processing. Nevertheless, no other species corresponding to IP degradation products were detected, demonstrating that no significant cell lysis or degradation of IP occurs during the process (S1 Fig).

This high-level secretory production of IP in the culture supernatant compares favorably to the highest yields previously published of 1.5 to 3.84 g L^-1^ [6,7,11] and is significantly higher than others published which are in the range of 0.18 to 0.3 g L^-1^ [12–15].

### Clarification of bioreactor’s cell culture in tangential flow filtration system

In order to separate cells from the culture broth a tangential flow stacked plate membrane device (small scale Prostak^™^ prototype, Merck Millipore) with open feed channels in the tangential flow filtration (TFF) system was utilised [16]. In this way we circumvented the classical clarification procedure that involves a combination of centrifugation and depth filtration. Prostak^™^ device can handle feed streams with high viscosity and high particulates/solids contents, due to the absence of turbulence-promoter screens between membranes plates, which are usually present in other typical TFF devices in the cassettes. Fermentation broth with a cell concentration of 34% w/v (9.5 kg of wet cell weight in 28 litres of harvested culture broth) with a turbidity of >1000 NTU was used as this presented the best feed stream candidate, being more concentrated of the 3 fermentations performed, to explore the concentration capabilities of the Prostak^™^ device.

**Fig 1:**
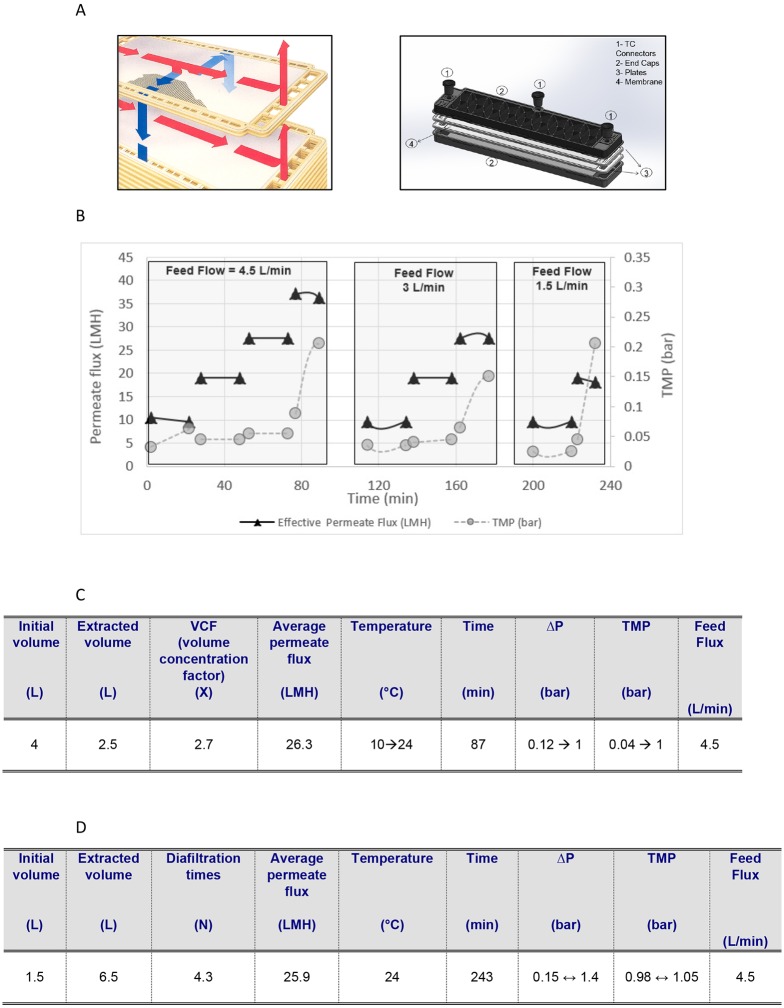
(A) Left panel: Flows diagram for the commercial Prostak^™^ devices (red line for feed-retentate and blue line for permeate). Right panel: exploded view of small scale Prostak^™^ prototype (2 stak-0.063 m^2^). (B) Critical Permeate Flux Study (CPF) with the small scale Prostak^™^ prototype Tangential Flow Filtration system was run at the three cross flux settings of 4.5 L/min, 3 L/min and 1.5 L/min, with resulting critical permeate fluxes of, respectively, 37 LMH (L/m2/h), 28 LMH and 19 LMH. (C) Concentration step of the TFF clarification procedure with the critical data provided. (D) Diafiltration step of the TFF clarification procedure with the critical parameters provided.

The clarification experiment was run using a small scale Prostak^™^ prototype module from Merck Millipore (Fig 1A), which incorporates Durapore 0.22 μm membrane (PVDF) and offers the benefit of a small effective area (EFA) requiring lower feed recirculation rates than those associated with the smallest standard devices. This operational advantage translates into easier operations at handling small lab-scale experiments, with flexibility to address the recent growing demand for clarification of feed streams with ever increasing cells titres. The experiment was run on a 4 L sample of *Pichia pastoris* fermentation broth containing IP. The optimization study (Critical Permeate Flux study—CPF) was run in total recirculation mode, to set initial hydraulic conditions for the next phases of concentration and diafiltration. We initially determined the permeate flux value which destabilizes the TFF system (critical permeate flux), when the device’s transmembrane pressure (TMP) starts to increase fast, to reach as a rule-of-thumb a value equal to 1.5–2 times or more the initial TMP [17]. The study was run at three cross flux settings of 4.5 L/min, 3 L/min and 1.5 L/min, with resulting critical permeate fluxes of 37 LMH (L/m^2^/h), 28 LMH and 19 LMH respectively (Fig 1B). Concentration and diafiltration were then run at an average permeate extraction rate of 26.3 LMH and 25.9 LMH respectively, comparable to around 70% of the critical permeate flux to work with stable TMPs (Fig 1C and 1D). The concentration was stopped at volume concentration factor (VCF) of 2.7X, as at this stage TMP had reached 1 bar and it was necessary to avoid potential plugging of the Prostak^™^ module. Clarification was successful with good visual clarity of permeate (turbidity of 46 NTU against an initial of >1000). Permeate volume was 2.5 L and contained around 80% of total IP as determined by analytical RP-HPLC. This is close to the theoretical value of 87%, as in the 4 L of culture broth 1.13L corresponds to cell volume and just 2.87 L to supernatant.

The concentrated harvest (1.5 L) was then subjected to diafiltration with Milli-Q grade water to complete the recovery of IP. Diafiltration volume was 6.5 L and contained around 20% of IP as determined by analytical RP-HPLC. Therefore, the clarification method performed showed complete insulin precursor recovery (from 4L culture broth) in a total volume of 9L.

Overall, no significant flux reduction during concentration was observed, the permeate flux during diafiltration was stable, TMP change remained constant over time from diafiltration cycle to cycle supporting constant conditions providing indirect evidence for no cell breakage or shear damage during the run. Indeed, analytical RP-HPLC showed no additional peaks detected post TFF in comparison to the sample pre TFF (S2 Fig panels A, B and C). As the sample had a clear stream it was directly loaded onto the chromatographic column without any secondary clarification step. These results suggest that use of the Prostak^™^ device with 0.22μm membrane in the tangential flow filtration is a promising technique for clarification of the high-density bioprocess culture broths.

### IP purification and concentration

In the optimisation of downstream process optimisation, we sought to increase efficiency of the capture step for IP. In addition to the binding strength, elution behaviour of IP was studied in order to obtain a high IP titer that could be utilised directly in the transpeptidation reaction, without need for the further concentration or lyophilisation.

**Fig 2:**
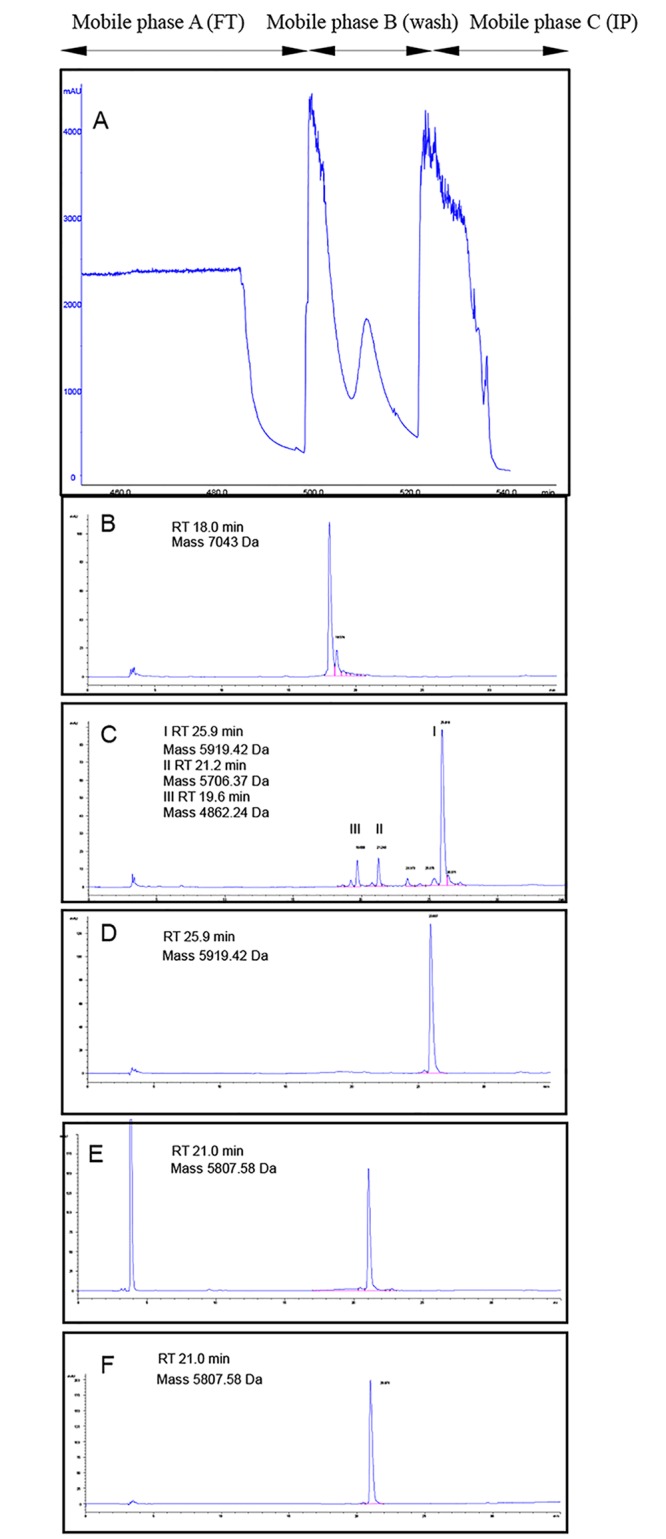
(A) Toyopearl Giga cap S-650 M cation-exchange chromatography of IP. IP was eluted from the column with the mobile phase C: 20 mmol L^-1^ NaOAc, pH 4.0, EtOH 50%, NaCl 0.5 mol L^-1^. RP-HPLC profiles of insulin species from each step of the human insulin production process (panels B-F). (B) RP-HPLC profile of the insulin precursor after cation exchange chromatography. IP has experimental molecular mass 7043 Da (calculated average mass is 7042.04) and retention time 18.0 minutes. (C) RP-HPLC profile of insulin species after the transpeptidation reaction. Insulin ester-H-Thr(tBu)-OtBu is eluted at retention time 25.9 minutes (experimental and calculated average mass 5919.42 Da), insulin species cleaved at B29 without threonine ester are eluted at retention time 21.2 minutes (experimental and calculated average mass 5706.37 Da) and insulin species cleaved at B22 are eluted at retention time 19.6 minutes (experimental and calculated monoisotopic mass 4862.24 Da). (D) RP-HPLC profile of insulin ester-H-Thr(tBu)-OtBu after the reversed-phase chromatography purification. (E) RP-HPLC profile of deprotection reaction mixture where human insulin has a retention time 21.0 minutes (experimental and calculated average mass 5807.58 Da). (F) RP-HPLC profile of purified human insulin (after the last reversed-phase chromatography). The purity of the human insulin was at least 98%.

The material recovered by TFF was loaded directly onto two different cation exchange resins for purification, Sepharose SP Fast Flow and Toyopearl GigaCap S-650 M. Toyopearl GigaCap S-650 M showed a higher loading capacity for IP (110 g L^-1^) along with IP binding at relatively high conductivity (15 mS cm^-1^) in comparison to Sepharose SP Fast Flow matrix (36 g L^-1^ loading capacity at the conductivity 8 mS cm^-1^). We optimised the elution buffer composition in order for it to be similar to the mixture of water and organic solvents typically used in transpeptidation and to reach the purification yields of more than 95% (Table 1). IP was eluted in 20 mmol L^-1^ NaOAc, pH 4.0, EtOH 50%, NaCl 0.5 mol L^-1^ buffer with the concentration of 80 g L^-1^ from Toyopearl GigaCap S-650 M (Fig 2A, mobile phase C—peak 3 and Fig 2B) and at 25 g L^-1^ from Sepharose SP Fast Flow resin. Analysis of the 3 peaks coming out from Toyopearl GigaCap S-650M column (Fig 2A), from left to right, was performed by analytical RP-HPLC and show that IP is present only in peak 3 (S2 Fig panels D to G). Storage of IP in the elution buffer at +4°C did not compromise quality of IP as determined by RP-HPLC and mass spectrometry even after 6 months (data not shown).

**Table 1:** Overview of insulin production and purification process from *Pichia pastoris* fermentation culture.

Steps	Insulin species	Molecular Mass (Da) ^b^	mg ^a^	mmol ^a^	Recovery (%) ^a^	Purity (%) ^a^
Culture supernatant	Insulin precursor	7043.04	2520	0.357	100	n.d.
Cation exchange	Insulin precursor	7043.04	2500	0.355	99	n.d.
Transpeptidation	Insulin ester	5919.42	1575	0.266	75	60
Reversed-phase/ Crystallisation	Human insulin	5919.42	1240	00.209	58	90
Deprotection	Human insulin	5897.58	1260	0.216	60	90
Reversed-phase	Human insulin	5807.58	1060	0.182	51	98

IP was recovered from the culture supernatant by TFF. Cation exchange chromatography was performed on Toyopearl GigaCap S-650 M resin and reversed-phase chromatography on PLRP-S 300Å 8μm resin. Molecular mass of insulin precursor, insulin intermediates and human insulin is the experimental average mass that corresponds to the calculated average for each specimen.

^a^ Recovery and purity was determined by RP-HPLC. Recovery was calculated from the protein amount determined in mmol.

^b^ Molecular mass of insulin species was determined by mass spectrometry. n.d., not determined

We therefore propose that the use of Toyopearl GigaCap S-650 M resin coupled to the described elution strategy as a novel procedure to capture IP at high load with elution at high concentration (~80 mg/ml) in a solvent mixture suitable for transpeptidation without intermediate step of diafiltration/buffer exchange. The results obtained here propose Toyopearl GigaCap S-650 M resin and a novel elution strategy as a promising high-capacity IP capture step on cation exchange media with an elevated step yield.

### Conversion of IP to human insulin

The insulin precursor was converted enzymatically into insulin ester via transpeptidation [18–20] in the presence of trypsin and an excess of O-t-butyl-L-threonine t-butyl ester (H-Thr(tBu)-OtBu) acetate salt. Trypsin mediates cleavage of the N-terminal spacer peptide and the Ala-Ala-Lys connecting linker peptide and the transesterification reaction where threonine ester is added to B-29 residue as previously described [6]. We developed cation exchange chromatography elution conditions such that the concentrated IP eluted in the organic solvent could be directly used in the transpeptidation. In this manner we omitted a slow and expensive lyophilization step, which can also be a source of batch-to-batch inconsistency. In particular, with the Toyopearl GigaCap S-650 M resin we were able to obtain high concentration of IP (80 g L^-1^) which allowed further reduction in the amount of the most expensive reagents in the transpeptidation reaction, namely trypsin and H-Thr(tBu)-OtBu.AcOH. The chosen combination of organic solvent, temperature, pH and amount of threonine and H-Thr(tBu)-OtBu was based on equilibrium between the cost and the yield of insulin ester obtained in the transpeptidation reaction. There are three main species formed during transpeptidation, which can be detected by analytical RP-HPLC (Fig 2C). Insulin ester-Thr(tBu)-OtBu was eluted at RT = 25.9 min, IP cleaved at B29 without threonine ester was eluted at RT = 21.2 min and IP cleaved at B22 was eluted at RT = 19.6 min. The IP is transformed into insulin ester with a yield of 75% (Table 1). Yields of up to 90% were obtained with the higher amounts of trypsin and H-Thr(tBu)-OtBu.AcOH used, with consequent increase in process cost. Insulin ester-Thr(tBu)-OtBu was further purified using preparative reversed-phase separation. We selected the resin PLRP-S 300Å 8μm resin (Agilent Technologies) that provides scalability from analytical separations to preparative columns and bulk media (Fig 2D, Table 1). After crystallization in the presence of zinc ions, insulin ester-Thr(tBu)-OtBu was converted into human insulin in the reaction of deprotection (removal of the tertiary butyl group from threonine in position B30) (Fig 2E). Finally, human insulin was purified by preparative RP-HPLC, on the PLRP-S 300A 8μm media (Agilent Technologies) as described in the Materials and Methods section. Recombinant human insulin purity was > 98% as determined by analytical RP-HPLC, fulfilling the European Pharmacopoeia requirements of purity (Fig 2F). The total yield of this newly developed simple, innovative and industrially acceptable process was 51% (Table 1).

### Recovery of excess of H-Thr(But)-OtBu from transpeptidation reaction

**Fig 3:**
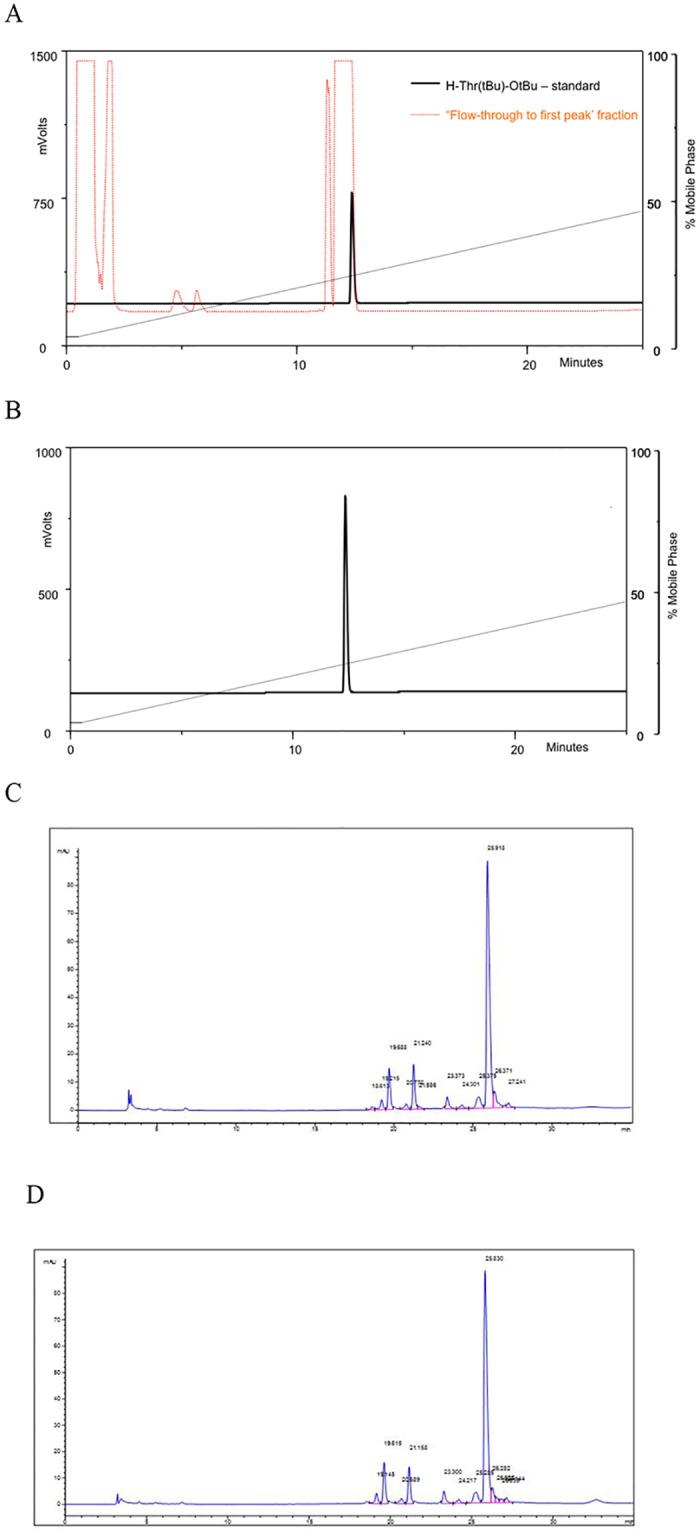
(A) Detector traces (Evaporative Light Scattering Detector) from RP-HPLC separation of H-Thr(tBu)-OtBu standard and flow-through to first protein peak fraction from post transpeptidation purification. (B) Detector traces (Evaporative Light Scattering Detector) from RP-HPLC separation of the recovered H-Thr(tBu)-OtBu from transpeptidation reaction of IP. Retention time of H-Thr(tBu)-OtBu is 12.36 min and the purity is 99%. **(C)** RP-HPLC profile of insulin species after transpeptidation reaction with H-Thr(tBu)-OtBu. (D) RP-HPLC profile of insulin species after transpeptidation reaction with extracted H-Thr(tBu)-OtBu. No difference in efficiency of the transpeptidation reaction was observed.

As H-Thr(tBu)-OtBu reagent, used in excess in the transpeptidation reaction, is one of the most costly reagents in the whole process, we evaluated the feasibility of recovering the non-reacted reagent by water organic solvent extraction. As described above, transpeptidation reaction mixture was applied on the PLRP-S 300A column in order to perform a preparative reverse phase chromatography. We collected the flow-through until the first protein peak fraction and proceeded with the liquid-liquid extraction (aqueous/organic phases) of residual H-Thr(tBu)-OtBu. Firstly, we performed an analytical RP-HPLC with the sample and standard in order to confirm the presence of the H-Thr(tBu)-OtBu reagent. H-Thr(tBu)-OtBu was detected in the collected fraction via analytical RP-HPLC at 214nm with Evaporative Light Scattering Detector (Fig 3A). As described in material and methods, we used a mixture of ethyl acetate and diethyl ether in the presence of salts to extract H-Thr(tBu)-OtBu. The yield of the recovered H-Thr(tBu)-OtBu was 80% and purity was 99% (Fig 3B). Electrospray mass spectrometry analysis of the eluted peak identified the molecule with a mass of 232.2 Da that corresponds to H-Thr(tBu)-OtBu. Extracted H-Thr(tBu)-OtBu was tested in a new transpeptidation of insulin precursor with no difference in comparison with the standard H-Thr(tBu)-OtBu (Fig 3C and 3D). Thus, we have developed an easy, cheap and an efficient method for H-Thr(tBu)-OtBu extraction from the transpeptidation reaction mixture which significantly reduces overall process cost without affecting the yield.

## Discussion

We have previously described a two-step fed-batch *Pichia* fermentation resulting in high-level secretory production of IP [6]. Methanol concentration during the induction phase was optimised and constantly controlled during the whole cultivation process. In this manuscript, we elaborate a set of industrially acceptable process steps for human insulin production with emphasis on simplicity, efficacy and cost reduction. We simplified the procedure and developed fed-batch fermentation with no need for constant in-process methanol concentration measurement that yielded 2.26 g L^-1^ of IP. In respect to IP recovery, although disk stack centrifugation is typically capable of managing high concentrations of cells in the feed, it is often limited in its ability to produce a clear product. High numbers of small particles are not efficiently removed by centrifugation and these may aggravate downstream chromatography creating the need for a secondary clarification step [21]. Tangential-flow filtration with microfiltration membranes has become a widespread technique for bioprocess culture broth clarification [22]. TFF devices with microfiltration cut-offs usually offer relatively low permeate extraction rates in clarification applications and in this study we show that a small scale Prostak^™^ prototype is no exception, with an average permeate fluxes of 26 LMH. However, when such TFF systems are optimized they represent a robust technology for clarification of feed streams with high solids content. Indeed, Prostak^™^ cartridge based TFF has been employed for clarification of different vaccines secreted from bacteria and successfully applied to pilot scale recovery [23–25] and inclusion bodies clarification from bacteria cell lysates [26].

On the basis of the test results reported here, the use of a 0.84 m^2^–10-stack Prostak^™^ module with the Durapore 0.22μm would allow clarification of 30 L fermentation culture broth of *Pichia pastoris* in four hours. The selection of 0.22μm tight membranes of facilitates removal of lipids and other colloids, thereby eliminating the need for further filtration devices. Moreover, we did not observe cell breakage and shear damage indicating that the Prostak^™^ cassette is a good alternative for the clarification step, cutting the clarification process down to just one step. As Prostak^™^ filters have been designed for industrial applications they are available in different dimensions facilitating the process of scaling-up. Moreover, the modularity of the device offers a significant advantage in this process.

We subsequently, we focused our attention on the optimization of the purification process of the IP, establishing a method of water/ethanol elution of IP from the cation exchange chromatography at a high titer. It has already been demonstrated that the use of an organic solvent may reduce or eliminate the formation of IP dimers and or hexamers, thus providing an appropriate environment for further IP processing [27]. Moreover organic solvents such as ethanol enhance the catalytic properties of enzymes, probably due to the increased stability of the enzyme native secondary structure elements in these mixtures [28–31]. In this way we were able to avoid a subsequent step of diafiltration/buffer exchange or freeze-drying, usually necessary to obtain highly concentrated IP in the mixture of water and organic solvents used in the enzymatic transpeptidation reaction. This is particularly advantageous as lyophilisation (freeze-drying) is a slow and expensive process raising issues of product activity, stability, batch consistency and repeatability. Bypassing the freeze-drying step in insulin production process eliminates these setbacks. Interestingly, use of Toyopearl GigaCap S-650 M, interestingly, has been shown to a suitable as a capture step for monoclonal antibodies with ≥90 g/L dynamic binding capacity operating at high linear flow rates up to 900 cm/h. Therefore this resin is readily scalable with significant cost savings in comparison to the traditionally used protein A resins [32,33].

We optimized all transpeptidation reaction conditions including as temperature, reaction time, enzyme concentration, pH and concentration of organic solvents in order to improve the digestion conversion rate and to develop a cost-efficient process, reaching a 75% recovery. Moreover, the second freeze-drying step was omitted whilst introducing an efficient and inexpensive crystallization step. Ultimately, further reduction in the process costs comes from the recovery of the excess of H-Thr(tBu)-OtBu from the transpeptidation reaction mixture, that we successfully show can be extracted and re-utilised.

All the developments described in this manuscript represent a progress towards an improved large scale manufacturing of human insulin with reduced cost and no consequences on the final insulin product quality.

## Supporting Information

S1 Fig Analysis of the culture supernatant from bioreactor during 6 days of methanol induction. A. RP-HPLC profiles of culture supernatant from bioreactor during 6 days of methanol induction. Insulin precursor peak has a retention time around 18 minutes. B. Amino acid sequence (63 aa) of Insulin precursor. C. Deconvoluted molecular mass spectrum of the whole supernatant at day 6 analysed by mass spectrometry (ESI-MS). Experimental molecular masses detected were 7043.04 Da, 6713.27 Da, 6584.20 Da, 6512.50 and 6384.00 Da corresponding to insulin precursor species with different length of N-terminal extension EEAEAEAEPK. No other species corresponding to IP degradation products were detected
(PDF)

S2 Fig Analytical RP-HPLC analysis of the sample pre TFF (Panel A), TFF permeate (Panel B) and TFF diafiltrate (Panel C). Main peak in the chromatograms is Insulin precursor with the retention time of around 18 minutes. Signals of retention time before 5 min correspond to the flow-through. No additional peaks are detected in the sample post TFF in comparison to the sample pre TFF giving an evidence of no cell breakage during TFF. Panels D-G. Analysis of peaks coming out from Toyopearl GigaCap S-650M column, from left to right (Fig 2 panel A) by analytical RPHPLC. Panel D Flow through; Panel E Peak No.1; Panel F Peak No.2; Panel G Peak No.3 Insulin precursor).
(PDF)
