# Supplementary material for: A Simplified and Efficient Process for Insulin Production in Pichia pastoris
Source: PLoS One. 2016 Dec 1;11(12):e0167207. doi: 10.1371/journal.pone.0167207 (PMC5131935; doi:10.1371/journal.pone.0167207)
Supplement: S1 Fig — A. RP-HPLC profiles of culture supernatant from bioreactor during 6 days of methanol induction. Insulin precursor peak has a retention time around 18 minutes. B. Amino acid sequence (63 aa) of Insulin precursor. C. Deconvoluted molecular mass spectrum of the whole supernatant at day 6 analysed by mass spectrometry (ESI-MS). Experimental molecular masses detected were 7043.04 Da, 6713.27 Da, 6584.20 Da, 6512.50 and 6384.00 Da corresponding to insulin precursor species with different length of N-terminal extension EEAEAEAEPK. No other species corresponding to IP degradation products were detected (PDF) [file pone.0167207.s001.pdf]

Figure S1

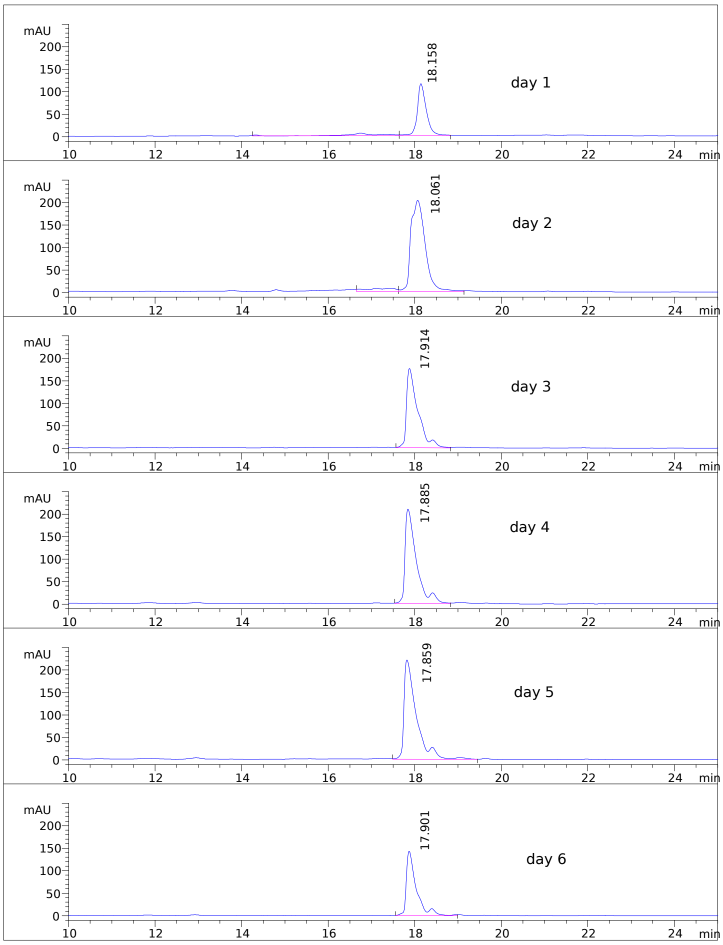

A

EEAEAEAEPK – FVNQHLCGSHLVEALYLVCGERGFFYTPK -- AAK-

1--------------------------10----------------------------------20----------------------------30----------------------------------40-------

N-terminal extension | Insulin B-chain | Linker

-GIVEQCCTSICSLYQLENYCN

-----------------------50---------------------------60------ 63

Insulin A-chain

B

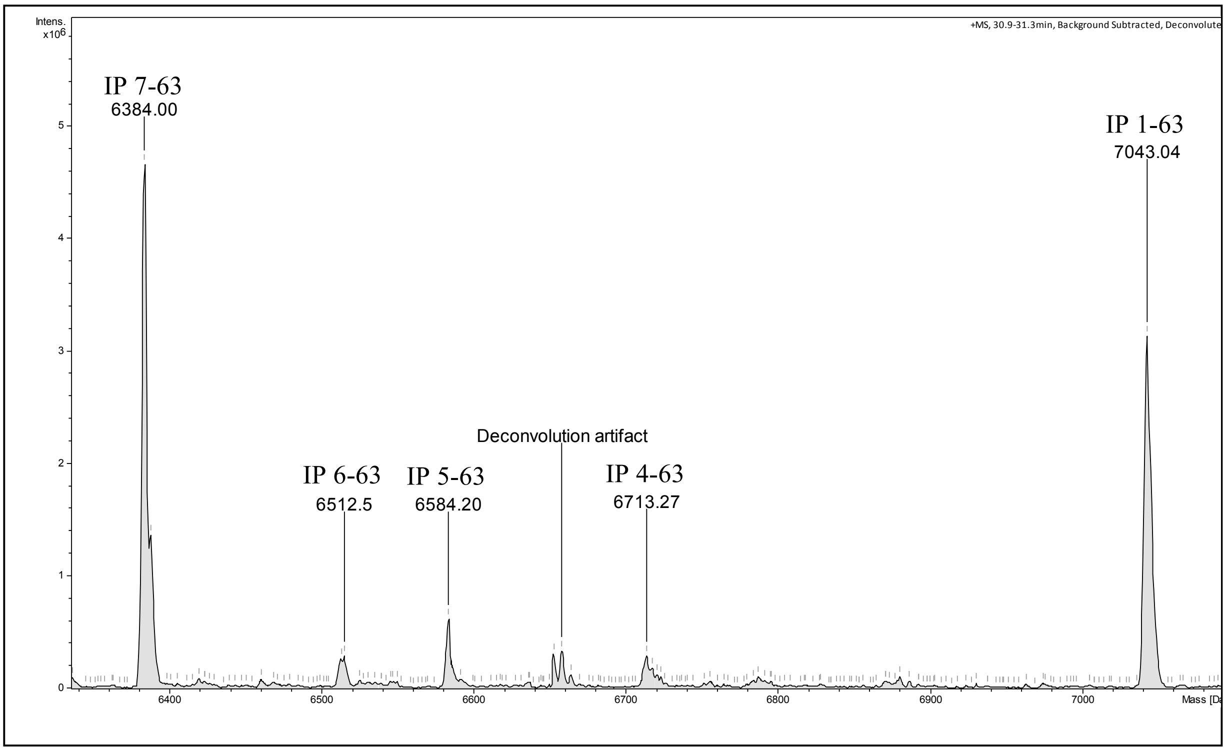

C

Figure S1. Analysis of the culture supernatant from bioreactor during 6 days of methanol induction. A. RP-HPLC profiles of culture supernatant from bioreactor during 6 days of methanol induction. Insulin precursor peak has a retention time around 18 minutes. B. Amino acid sequence (63 aa) of Insulin precursor. C. Deconvoluted molecular mass spectrum of the whole supernatant at day 6 analysed by mass spectrometry (ESI-MS). Experimental molecular masses detected were 7043.04 Da, 6713.27 Da, 6584.20 Da, 6512.50 and 6384.00 Da corresponding to insulin precursor species with different length of N-terminal extension EEAEAEAEPK. No other species corresponding to IP degradation products were detected.
