# Supplementary material for: A Simplified and Efficient Process for Insulin Production in Pichia pastoris
Source: PLoS One. 2016 Dec 1;11(12):e0167207. doi: 10.1371/journal.pone.0167207 (PMC5131935; doi:10.1371/journal.pone.0167207)
Supplement: S2 Fig — Main peak in the chromatograms is Insulin precursor with the retention time of around 18 minutes. Signals of retention time before 5 min correspond to the flow-through. No additional peaks are detected in the sample post TFF in comparison to the sample pre TFF giving an evidence of no cell breakage during TFF. Panels D-G. Analysis of peaks coming out from Toyopearl GigaCap S-650M column, from left to right (Fig 2 panel A) by analytical RPHPLC. Panel D Flow through; Panel E Peak No.1; Panel F Peak No.2; Panel G Peak No.3 Insulin precursor). (PDF) [file pone.0167207.s002.pdf]

Figure S2

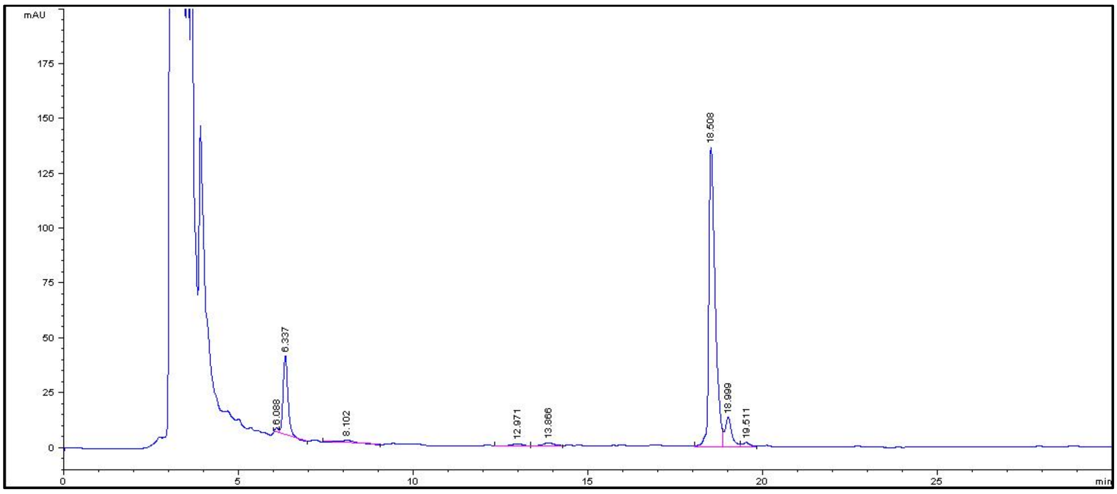

A

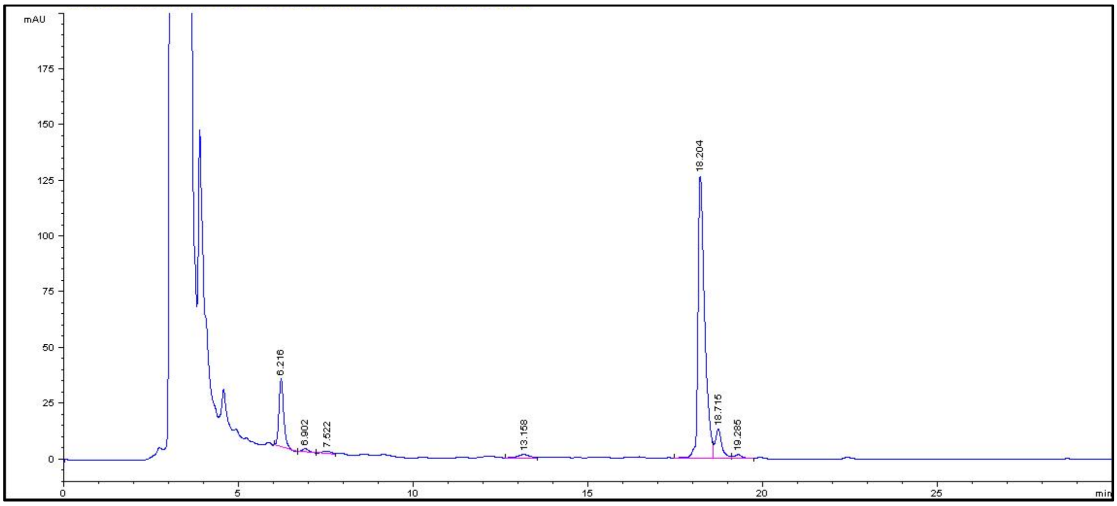

B

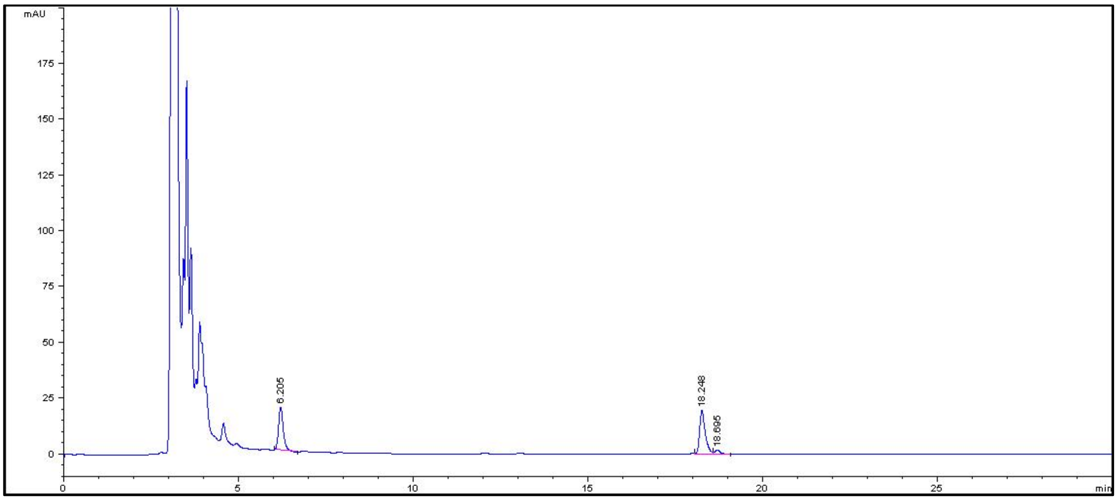

C

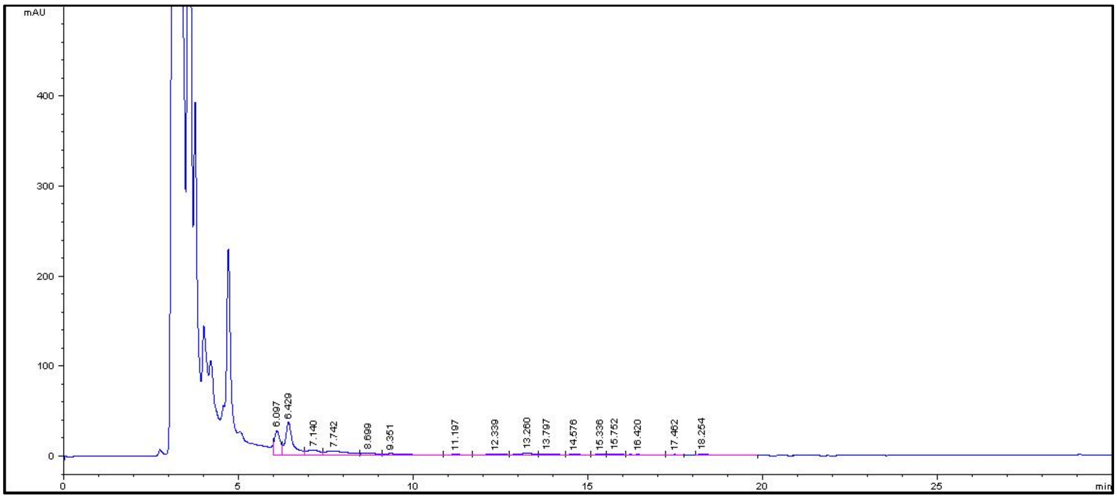
mAU
400
300
200
100
0
0
5
10
15
20
25
min
6.097
6.429
7.140
7.742
8.699
9.351
11.197
12.339
13.260
13.797
14.576
15.336
15.752
16.420
17.462
18.254

D

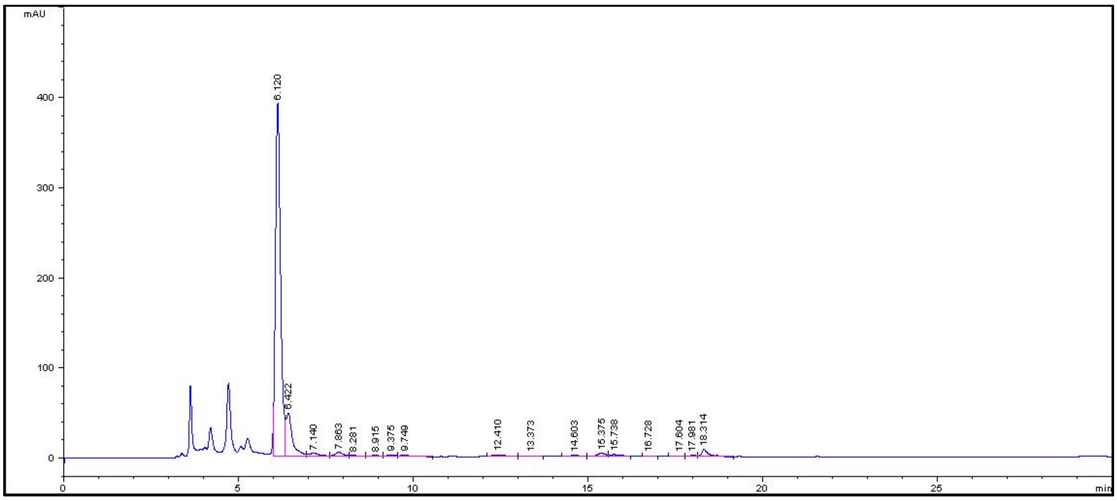
mAU
400
300
200
100
0
0
5
10
15
20
25
min
6.120
6.422
7.140
7.863
8.281
8.915
9.375
9.749
12.410
13.373
14.603
15.375
15.738
16.728
17.604
17.981
18.314

E

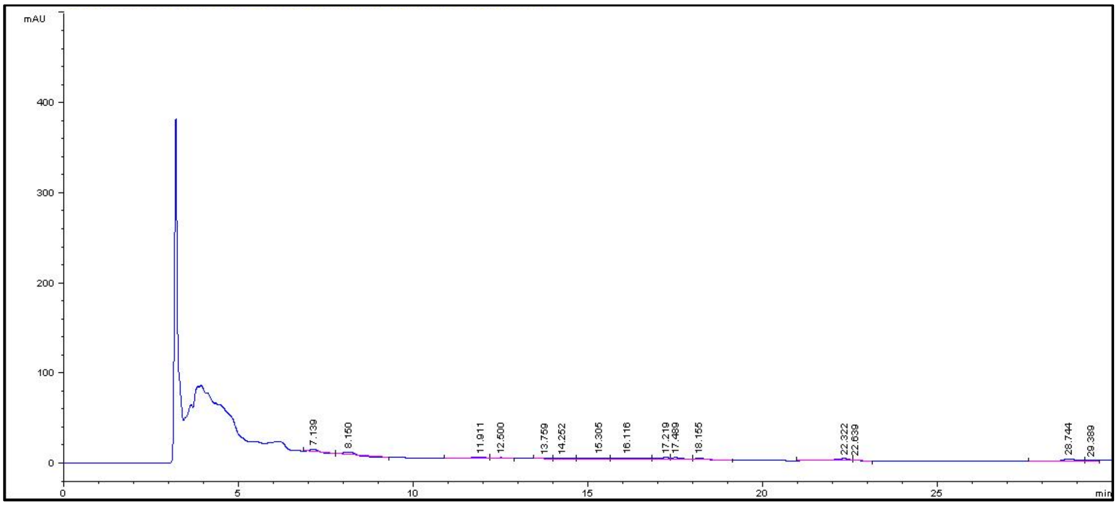
mAU
400
300
200
100
0
0
5
10
15
20
25
min
7.139
8.150
11.911
12.500
13.759
14.252
15.305
16.116
17.219
17.489
18.155
22.322
22.639
28.744
29.389

F

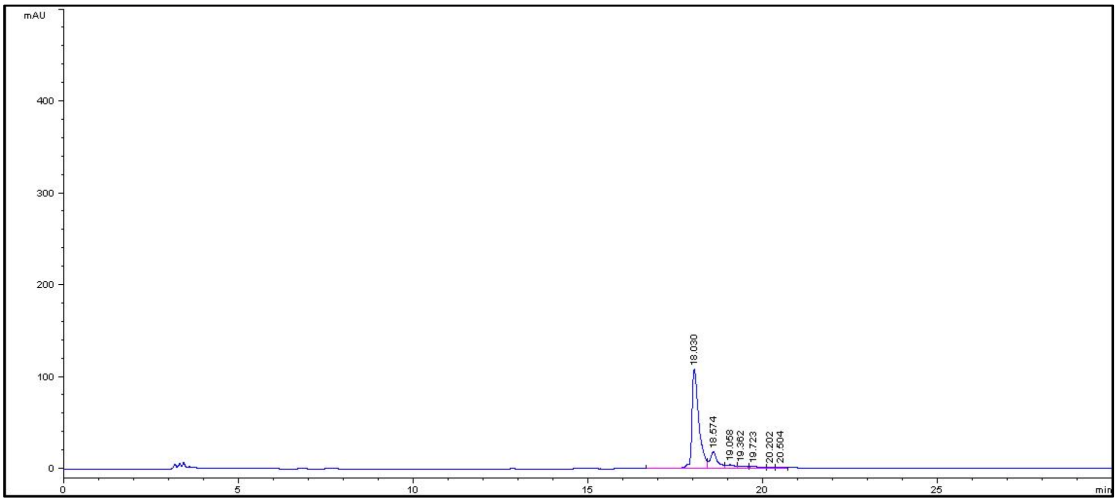

G

Figure S2. Analytical RP-HPLC analysis of the sample pre TFF (Panel A), TFF permeate (Panel B) and TFF diafiltrate (Panel C). Main peak in the chromatograms is Insulin precursor with the retention time of around 18 minutes. Signals of retention time before 5 min correspond to the flow-through. No additional peaks are detected in the sample post TFF in comparison to the sample pre TFF giving an evidence of no cell breakage during TFF. Panels D-G. Analysis of peaks coming out from Toyopearl GigaCap S-650M column, from left to right (Figure 2 panel A) by analytical RP-HPLC. Panel D Flow through; Panel E Peak No.1; Panel F Peak No.2; Panel G Peak No.3 Insulin precursor).
